# 46,XY Disorder of Sex Development due to 17-Beta Hydroxysteroid Dehydrogenase Type 3 Deficiency in an Infant of Greek Origin

**DOI:** 10.4274/jcrpe.4829

**Published:** 2018-02-26

**Authors:** Assimina Galli Tsinopoulou, Anastasios Serbis, Eleni P. Kotanidou, Eleni Litou, Vaia Dokousli, Konstantina Mouzaki, Pavlos Fanis, Vassos Neocleous, Nicos Skordis

**Affiliations:** 1 Aristotle University of Thessaloniki Faculty of Medicine, Department of Health Sciences, Papageorgiou General Hospital, 4th Clinic of Pediatrics, Thessaloniki, Greece; 2 The Cyprus Institute of Neurology & Genetics, Department of Molecular Genetics, Function & Therapy, Nicosia, Cyprus; 3 Paedi Center for Specialized Pediatrics, Division of Pediatric Endocrinology, Nicosia, Cyprus; 4 St. George’s University of London Medical School at the University of Nicosia, Nicosia, Cyprus

**Keywords:** Disorder of sex development, 17-β-hydroxysteroid dehydrogenase type 3 deficiency, HSD17B3 gene, androstenedione, testosterone

## Abstract

17-beta hydroxysteroid dehydrogenase type 3 (17βHSD-3) enzyme catalyzes the conversion of androstenedione (Δ4) to testosterone (T) in the testes of the developing fetus, thus playing a crucial role in the differentiation of the gonads and in establishing the male sex phenotype. Any mutation in the encoding gene (HSD17B3) can lead to varying degrees of undervirilization of the affected male, ranging from completely undervirilized external female genitalia to predominantly male with micropenis and hypospadias. We present here an infant who was referred to our clinic because of ambiguous genitalia at birth. Gonads were palpable in the inguinal canal bilaterally and no Müllerian structures were identified on pelvic ultrasound. Because of a low T/Δ4 ratio after a human chorionic gonadotropin stimulation test, a tentative diagnosis of 17βHSD-3 deficiency was made which was confirmed after genetic analysis of the HSD17B3 gene of the patient. The molecular analysis identified compound heterozygosity of two previously described mutations and could offer some further validation for the idea of a founder effect for 655-1;G→A mutation in the Greek population.

## What is already known on this topic?

17β-hydroxysteroid dehydrogenase type 3 (17βHSD-3) enzyme deficiency is a rare cause of ambiguous genitalia in XY neonates due to inadequate testosterone production that leads to undervirilization in utero.

## 

### What this study adds?

This study describes a neonate with ambiguous genitalia that proved to be a compound heterozygote for the gene responsible for 17βHSD-3 production and could offer some further validation for the idea of a founder effect for 655-1;G→A mutation in the Greek population.

## Introduction

The development of the male internal and external genitalia in a 46,XY fetus requires a complex interplay of several crucial genes, hormones and enzymes.At the time of fertilization the *chromosomal sex* is established, which in turn defines *gonadal sex* and in the final phase of development, *phenotypic sex* is established by the production and action of specific hormones (1).

Among these hormones, testosterone, together with its 5α-reduced end-product dihydrotestosterone (DHT), plays a crucial role in the development of internal and external genitalia of the male fetus. Testosterone is biosynthesized from cholesterol in five enzymatic steps, the last being the conversion of androstenedione (Δ4) to testosterone that takes place in the testes. This step of testosterone biosynthesis is catalyzed by the enzyme 17-βeta hydroxysteroid dehydrogenase type 3 (17βHSD-3). This enzyme is expressed solely in the testes and belongs to a large enzyme family called 17-β-hydroxysteroid dehydrogenase enzymes. Any damaging mutation in the *17βHSD-3* gene (*HSD17B3*) can cause a 46, XY disorder of sex development (DSD), i.e. a child with a 46,XY karyotype and atypical gonadal or anatomical sex. These mutations can be either homozygous or compound heterozygous and can cause variable 17βHSD-3 enzyme deficiency (2).

Here, we report the case of an infant who presented with ambiguous external genitalia and gonads that were bilaterally palpable in the inguinal region. A 46,XY karyotype was found and 17βHSD-3 deficiency was suspected after measuring a lower than normal testosterone/Δ4 ratio, after a human chorionic gonadotropin (hCG) stimulation test. *HSD17B3* gene was genetically analyzed confirming the diagnosis and further validating the idea of a founder effect for 655-1;G®A mutation in the Greek population.

The concept of the founder effect is as follows. When a new population is established in a new area by a very small number of individuals, the descendants of this population will show loss of genetic variation. This can be expressed by specific gene mutations being present in the given population, in a much higher frequency than the rest of the world.

## Case Report

The patient was the third baby born to a healthy, non-consanguineous couple of Greek origin. He was born at 37 weeks of gestation by caesarean section with a birth weight of 2860 g and with no perinatal problems. He was referred to our hospital at the age of 23 days due to his ambiguous genitalia at birth. Before referral, his karyotype was determined as 46,XY.

Institutional review board approval was obtained and both the child’s parents signed an informed consent in accordance with the national laws. Parents were verbally informed during the investigation regarding the purpose of the study.

**Figure 1:**
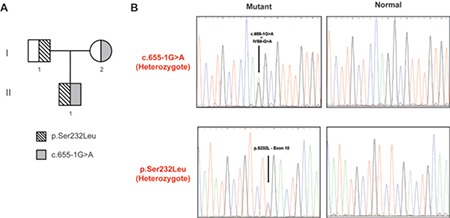
Identification of HSD17B3 mutations associated with 17-β-Hydrogenase type 3 deficiency. Pedigree of the family with HSD17B3; p.Ser232Leu and c.655-1G>A mutations. Grey shading indicates the presence of the c.655-1G>A mutation and filled line shading indicates the presence of the p.Ser232Leu mutation (A). Part of the sequencing electropherograms of the HSD17B3 gene showing the heterozygous mutations (p.Ser232Leu, c.655-1G>A) detected in individuals with HSD17B3 deficiency. The non-mutated sequences (normal) are depicted (B)

On physical examination, he had a phallus-like structure of 1.5 cm, perineoscrotal hypospadias, a perineal blind vaginal pouch and posterior labioscrotal fusion (Figure 1). Two masses, assumed to be the gonads, were palpable in the inguinal canal bilaterally. The rest of the physical examination was normal. Complete blood count and urine analysis were normal. An ultrasound examination confirmed the testicular structure of the masses in the inguinal canals and showed the absence of female internal genitalia.

Baseline LH, FSH, ACTH and cortisol concentrations, measured upon admission, were 12.1 IU/L (0.02-7.0 IU/L), 0.58 IU/L (0.16-4.1 IU/L), 24 pg/dL (5-90 pg/dL) and 11.20 µg/dL (2.8-23 µg/dL), respectively (Table 1). Androgen levels were as follows: Δ4: 10.68 ng/dL (10-37 ng/dL), testosterone: 25 ng/dL (60-400 ng/dL), DHT: 11 ng/dL (12-85 ng/dL) (all normal values given are age-specific). Since baseline hormonal values of various causes of 46,XY undervirilization can significantly overlap, the patient underwent a three-day hCG stimulation test. The results of the test showed a significant increase in Δ4 compared to testosterone and the low testosterone/Δ4 ratio in combination with a normal testosterone/DHT ratio was constistent with a diagnosis of 17βHSD3 deficiency (3).

Mutation analysis of the *HSD17B3 *gene in the patient was performed using real-time PCR and identified compound heterozygosity of the previously reported missense p.Ser232Leu and the splice junction variant 655-1;G®A (Figure 1). Further genetic analysis in both parents revealed the p.Ser232Leu mutation to be inherited from the father and the 655-1;G®A from the mother...” (i.e. the double space after A should be corrected).

**Figure 2:**
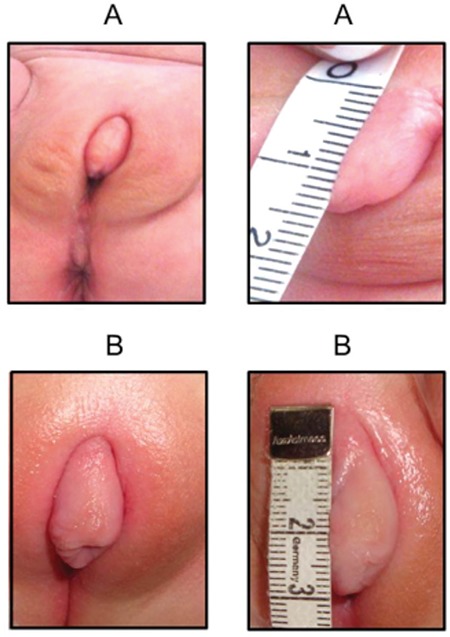
Phallus size in cm before (A) and after (B) administration of testosterone

After discussion with the parents, the decision to raise the child as male was reached. A clinical trial of three doses of 25 mg testosterone enanthate intramuscularly, at four-week intervals was initiated which resulted in an increase in penis size from 1.5 cm to 3 cm (Figure 2). It was proposed that surgical repair of the undescended testes and for correction of the hypospadias be delayed until an appropriate age.

## Discussion

DSD is a group of congenital conditions in which development of chromosomal, gonadal or anatomical sex is atypical. Its incidence is 1 in 5000-5500 (0.018%) (4). The most recent consensus statement on management of intersex disorders classifies DSD in three major categories: sex chromosome DSD, 46,XX DSD and 46,XY DSD (5,6,7).

46,XY DSD comprise cases in which individuals with male chromosomal sex (XY) have atypical gonadal or anatomical sex. 46,XY DSD can be caused by several different defects most commonly involving defective androgen production and/or action. If the testes are normally developed in patients with 46,XY DSD, androgen insensitivity syndrome is usually the cause. Less frequently, 46,XY DSD can be the result of several different mutations involving any of the five enzymes responsible for the conversion of cholesterol to testosterone and its 5α-reduced end-product, DHT. Among these defects in testosterone production, the most frequent is a deficiency in the 17βHSD-3 enzyme (8).The 17βHSD-3 enzyme is expressed solely in the testes and belongs to a large group of enzymes, the 17-β-hydroxysteroid dehydrogenase enzymes. This family comprises 14 isoenzymes, all of which play a significant role in androgen and estrogen production (3).

17βHSD-3 enzyme deficiency leads to an autosomal recessive form of 46,XY DSD, which was first described in 1971 (9,10). Its precise incidence is unknown, but in a study from the Netherlands it was calculated to be around 1 in 147,000 live births with a heterozygote frequency of 1 in 135 (11). Among populations with a high intermarriage rate, such as the Arabs of the Gaza Strip, the incidence has been reported to be much higher, up to 1 in 100-300 (12,13). 

The clinical presentation of individuals with 17βHSD-3 deficiency can vary greatly. Affected males usually present with female external genitalia, fusion of the labia and blind ending vagina, with or without clitoromegaly (Sinnecker types 5 and 4). Less frequently, external genitalia can be ambiguous (Sinnecker type 3), or mainly male with hypospadias and micropenis (Sinnecker type 2) (11,14). Furthermore, phenotypic variation can occur between families with the same homozygous mutation and it seems clear that no specific phenotype is associated with a specific mutation (11). Our patient presented with ambiguous genitalia, with a phallus-like midline structure of 1.5 cm, hypospadias, perineal blind vaginal pouch, posterior labioscrotal fusion, while both his testes were localized in the inguinal canals (Sinnecker type 2 to 3).

Either homozygous or compound heterozygous mutations in the *HSD17B3* gene can cause variable 17βHSD-3 enzyme deficiency (4). This gene spans at least 60 kb, consists of 11 exons and is localized on the 9q22 chromosome. To our knowledge, 37 HSD17B3 gene mutations have been reported so far. These include duplication, amplification, intronic splice site and exonic deletion as well as missense and nonsense mutations (2). Some of these appear to be *de novo *mutations while others are apparently ancient. Since some of the latter have been identified with higher frequency in specific countries or populations, a founder effect has been suggested in several cases (11,15,16).

The phenomenon of founder effect is rather common in the Greek population, and several reports have documented examples of founder mutations in the Mediterranean basin (16,17,18,19). Many previously published reviews cluster the Greeks together with the Turks and the Syrians to show a founder effect regarding the 655-1;G®A mutation. According to these papers, this mutation might have spread through the above-mentioned populations during the Ottoman Empire, which extended across most of the Eastern Mediterranean and contributed to the racial admixture in this area (20).

So far, the only reported case of a Greek individual with 17βHSD-3 enzyme deficiency was a paper published in 1978 (21). It describes a 12-year old 46,XY patient reared as a female, with both parents being Greek immigrants residing in New York. The patient was diagnosed with 17βHSD-3 deficiency (termed 17-ketosteroid reductase deficiency at the time) and, later, he was identified to be a homozygote for the 655-1;G®A mutation (22). Our patient was a compound heterozygote for mutations in the *HSD17B3* gene, having inherited the mutant p.Ser232Leu allele from his father and the 655-1;G®A allele from his mother.

In order to have robust evidence of a founder effect in any population, a critical number of individuals presenting with the same mutation for a given genetic trait is needed. Unfortunately, we are describing a rare genetic disease in a rather small (11 million) population which is shown by the fact that our patient is only the second Greek individual ever described with 17βHSD-3 deficiency. This makes it very difficult to have more solid evidence.

Nevertheless, the specific mutation (655-1;G®A) appears to be present in high frequency in Turks and Syrians, populations that reside in areas that (together with present-day Greece) used to be part of the Ottoman Empire. Thus, it is plausible that these areas of the Eastern Mediterranean were populated by a rather small group of people, which caused some degree of loss of genetic variability. This would lead to an increased number of descendants with specific mutations in some of their genes, even though robust evidence for such founder effects, especially in rare conditions, are yet to be found.

The gender assignment of patients with 17βHSD-3 deficiencies can be quite challenging and necessitates the collaboration of several different medical professionals for the initial decision and the subsequent management plan and follow-up (23).If gonadectomy is withheld, individuals who are reared as females can present in late adolescence with marked virilization due to conversion of the increased amount of Δ4 to testosterone (24). Our patient was partly undervirilized at presentation and showed a good response to the clinical trial of testosterone enanthate as shown by the increase in penis size from 1.5 cm to 3 cm. His parents have been informed about the need for surgical correction of cryptorchidism (around the end of the first year) and hypospadias and microphallus (at least two surgical procedures in the first 2-3 years of life) and are currently satisfied with the development of their son.

## Figures and Tables

**Table 1:**
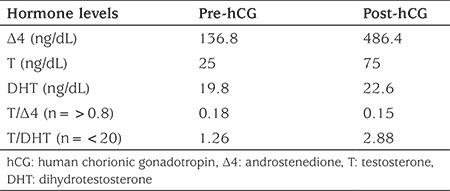
Serum androgen concentrations before and after human chorionic gonadotropin stimulation
